# Dramatic Shifts in Benthic Microbial Eukaryote Communities following the Deepwater Horizon Oil Spill

**DOI:** 10.1371/journal.pone.0038550

**Published:** 2012-06-06

**Authors:** Holly M. Bik, Kenneth M. Halanych, Jyotsna Sharma, W. Kelley Thomas

**Affiliations:** 1 Hubbard Center for Genome Studies, University of New Hampshire, Durham, New Hampshire, United States of America; 2 University of California Davis Genome Center, Davis, California, United States of America; 3 Department of Biological Sciences, Auburn University, Auburn, Alabama, United States of America; 4 Department of Biology, University of Texas, San Antonio, Texas, United States of America; American University in Cairo, Egypt

## Abstract

Benthic habitats harbour a significant (yet unexplored) diversity of microscopic eukaryote taxa, including metazoan phyla, protists, algae and fungi. These groups are thought to underpin ecosystem functioning across diverse marine environments. Coastal marine habitats in the Gulf of Mexico experienced visible, heavy impacts following the *Deepwater Horizon* oil spill in 2010, yet our scant knowledge of prior eukaryotic biodiversity has precluded a thorough assessment of this disturbance. Using a marker gene and morphological approach, we present an intensive evaluation of microbial eukaryote communities prior to and following oiling around heavily impacted shorelines. Our results show significant changes in community structure, with pre-spill assemblages of diverse Metazoa giving way to dominant fungal communities in post-spill sediments. Post-spill fungal taxa exhibit low richness and are characterized by an abundance of known hydrocarbon-degrading genera, compared to prior communities that contained smaller and more diverse fungal assemblages. Comparative taxonomic data from nematodes further suggests drastic impacts; while pre-spill samples exhibit high richness and evenness of genera, post-spill communities contain mainly predatory and scavenger taxa alongside an abundance of juveniles. Based on this community analysis, our data suggest considerable (hidden) initial impacts across Gulf beaches may be ongoing, despite the disappearance of visible surface oil in the region.

## Introduction

The *Deepwater Horizon* (DH) oil spill, commencing on April 20, 2010 and lasting for 89 days, represents one of the most dramatic anthropogenic impacts ever to hit the marine environment. Oceanic sediments harbour vast assemblages of microbial eukaryotes (nematodes, protists, fungi, etc.) that typically underpin key ecosystem functions such as nutrient cycling and sediment stability [1]. We presently have scarce knowledge of biogeographic patterns or community structure for these ‘invisible’ taxa in the Gulf of Mexico region, precluding any informed mitigation and remediation of sudden environmental impacts such as the DH oil spill. Determining post-spill foci and priorities in the Gulf of Mexico requires knowledge of historic patterns of biodiversity. Microbial eukaryotes inherently underpin all higher trophic levels, and thus, understanding the biological impact and subsequent recovery of these communities is critical for interpreting the long-term effects of the DH oil spill.

To investigate the impacts of heavy beach oiling that occurred during the DH spill, we utilized parallel marker gene and taxonomic approaches to characterize microbial eukaryote communities inhabiting beach sediments prior to and following shoreline oiling. Temporal replicates (May and September 2010) were collected from five sites around Dauphin Island and Mobile Bay, Alabama, with an additional post-spill site along a persistently oiled beach in Grand Isle, LA (Sept 2010). Our study focused on two diagnostic regions of the 18 S rRNA gene [2], independently amplified and sequenced on a 454 GS FLX Titanium platform. High-quality reads were clustered into Operational Taxonomic Units (OTUs) with stringency ranging from 95–99% pairwise identity and analysed within multiple bioinformatic pipelines to investigate and confirm biological inferences. Temporal community patterns were compared across all approaches utilized, including denoising of pyrosequencing data [3].

## Results

**Figure 1:**
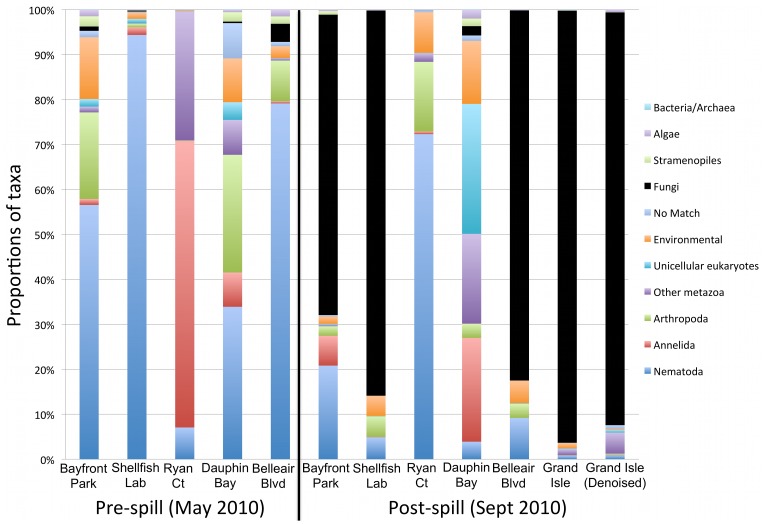
Pre-spill and Post-spill taxonomic comparisons of microbial eukaryote communities. Chart illustrating dominant microbial eukaryote taxa observed in pre-spill versus post-spill sample sites, with consistent nematode majority fractions giving way to fungal dominance in oil-impacted sediments. Proportions calculated from non-chimeric OTUs clustered in UCLUST at a 99% pairwise identity cutoff.

**Figure 2:**
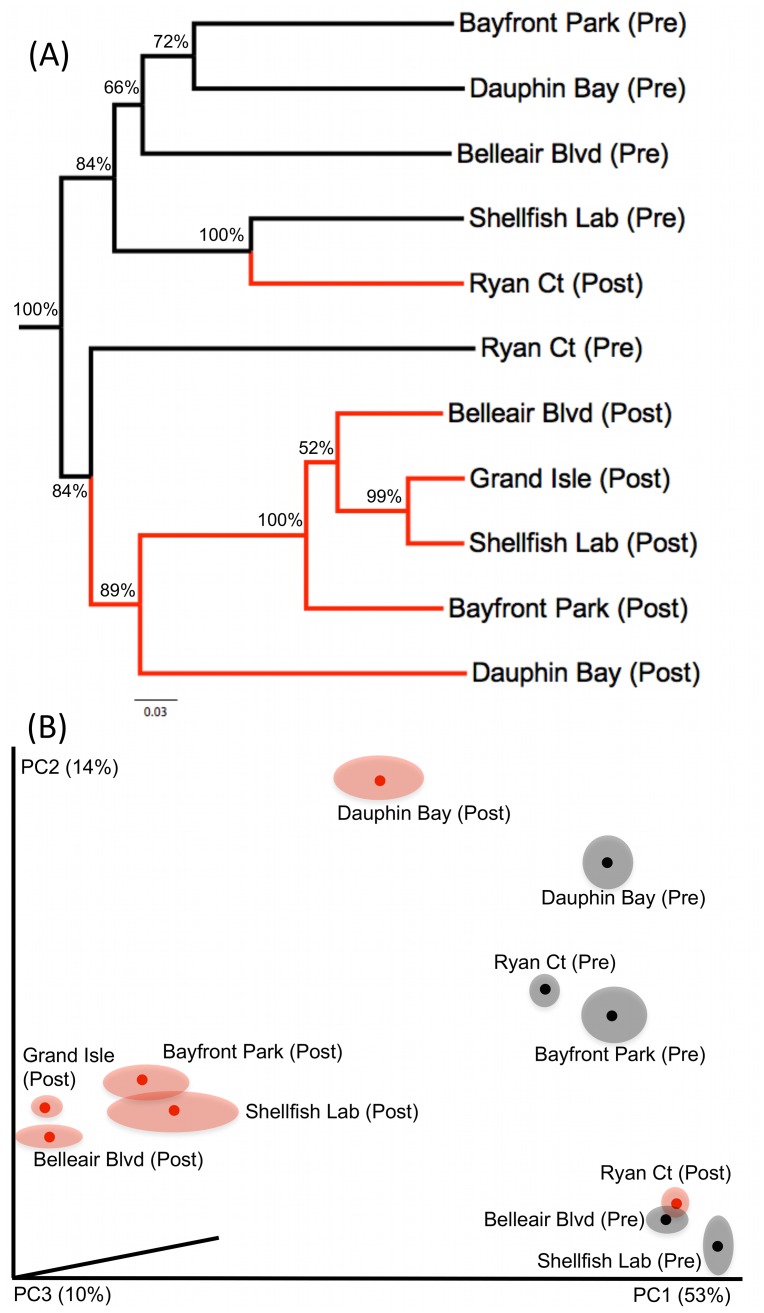
Phylogenetic beta diversity analysis of eukaryote communities conducted using the UniFrac distance metric [**5**] **.** (A) Jacknifed Cluster Analysis of non-chimeric OTUs (F04/R22 locus) clustered at 99% in UCLUST; support values >50% are reported. (B) Principal Coordinate analysis of non-chimeric OTUs (NF1/18 Sr2b locus) clustered under a 99% cutoff in UCLUST; 2D representation of a 3D plot (original 3D Kinemage files available in the accompanying Dryad data package) All analyses were conducted using weighted OTU datasets (normalized abundance values) sampled at an even depth across sites.

**Figure 3:**
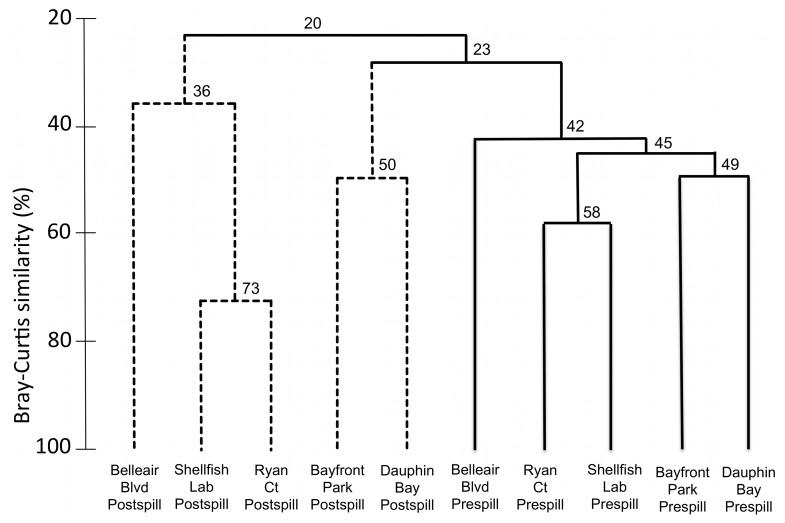
Bray-curtis similarity of morphologically identified nematode communities. Group average cluster analysis based on faunal resemblance; Bray-Curtis similarity of nematode genera calculated from pre-spill and post spill samples based on presence/absence of genera.

Marker gene datasets indicate a substantial shift in eukaryote communities between pre-spill and post-spill samples. Pre-spill sediments contain a high diversity of metazoan phyla, with the majority of sites showing an expected dominance of nematode taxa. In contrast, most post-spill sites are overwhelmingly dominated by fungi and show corresponding decreases in the number of metazoan taxa recovered. The observed patterns (Figure 1) were consistent regardless of the computational approach used to process data; community patterns were repeatedly recovered across a range of OTU clustering cutoffs in multiple pipelines, withstood denoising [4], and were not affected by three independent approaches for chimera checking/removal. Phylogenetically-informed beta diversity analyses (carried out using the UniFrac distance metric [5] implemented in the QIIME pipeline [6]) further support a distinction between pre-spill and post-spill eukaryote communities (Figure 2). None of our community structure analyses recovered a close relationship between paired pre- and post-spill replicates; instead, post-spill sites formed distinct groupings in both Principal Coordinate Analysis (PCoA) and Jackknife Cluster analysis. PCoA separated pre-spill sites across axes indicating diverse and divergent community assemblages inhabiting these beaches. In contrast, most post-spill sites converged into a singular cluster, driven by a common set of putatively oil-tolerant eukaryotes that appear to subsequently dominate affected sites. Our parallel morphological approach confirms a significant change in nematode assemblages. Pre-spill samples exhibited a high species richness and overall evenness in types of feeding strategies represented by the genera present, whereas post-spill samples showed significantly lower nematode richness, a bias towards predatory species and scavengers, and resident populations showing atypical abundances of juvenile stages. Based on presence/absence of morphologically identified genera, Bray-Curtis similarity analyses further supported similar community assemblages at post-spill sites (Figure 3). Lowered nematode abundances and similar taxonomic biases have been previously documented following hydrocarbon contamination in marine habitats [7], supporting altered functional roles within microbial eukaryote communities following oil exposure. In addition, some predatory nematode species are known for facultative utilization of alternate food sources [8] including direct uptake of dissolved carbon. This ability may confer a competitive advantage to opportunistic nematode species that are able to thrive by ingesting fungal prey or environmental carbon in oil-affected sediments.

Two distinct fungal community structures were recovered at post-spill sites: one assemblage dominated by *Cladosporium* OTUs (recovered at Shellfish Lab and Grand Isle), showing a close relationship to *C. cladosporioides* sequences in phylogenetic topologies, and a second assemblage dominated by OTUs in the fungal genus *Alternaria* (Belleair Blvd and Bayfront Park). Fungal taxon dominance may be dictated by the physical marine environment; *Alternaria* OTUs dominated in brackish Mobile Bay, while *Cladosporium* was recovered in higher-salinity sediments on the outer shores of Dauphin Island. These highly dominant post-spill OTUs appear as rare taxa in diverse pre-spill fungal assemblages, suggesting that oil-induced environmental stress may have favoured the rise of resilient, opportunistic species (able to capitalize on the large input of new resources). Although the diversity and ecological role of marine fungi is not well understood, previous evidence suggests that observed fungal assemblages denote a signature of crude oil in Gulf sediments. *Cladosporium* contains ubiquitous, opportunistic species that can extensively utilize hydrocarbon compounds and thrive in hostile, polluted conditions that appear to be intolerable for other marine fungi [9], [10]. Compared to many other fungi, marine *Altenaria* demonstrate increased activity of lignocellulose-degrading enzymes [11] that have been implicated in breakdown of industrial toxins [12], [13]. In addition to these dominant OTUs, we recovered a variety of fungi at post-spill sites (including OTUs phylogenetically related to *Apergillus*, *Acremonium*, *Acarospora*, *Rhodocollybia*, and *Rhizopus*) that rarely comprised a significant component of pre-spill fungal communities. A number of these marine groups have also been shown to metabolize hydrocarbon compounds [14], [15].

Two sites in our dataset did not follow typical pre/post spill community transitions observed at most locations: Ryan Court (located on the heavily developed Gulf facing shoreline of Dauphin Island) and Dauphin Bay (located on the bay side of the island facing the Alabama mainland). These two sites exhibited distinct post-spill community compositions (Figure 1) and appeared as outliers in beta diversity analyses (Figure 2). Following the oil spill, Ryan Court was dominated by nematode fauna (few taxa with a dominance of predators), a drop in Metazoa such as Annelids and Platyhelminthes, and low fungal abundances (primarily comprised of *Cladosporium* and *Alternaria*). Dauphin Bay exhibited a decrease in nematodes but retained a high overall proportion of metazoa, as well as maintaining a comparatively diverse fungal community. These notable shifts suggest some degree of disturbance resulting from hydrocarbon input and human-mediated beach cleaning activities, but appear to indicate less dramatic community change compared to other nearby sites. Over the course of the DH spill, the beach at Ryan Court was sporadically protected by heavy, waterborne barrier structures, while the Alabama facing Dauphin Bay site may have been somewhat sheltered from approaching oil slicks. Our results thus suggest a complex biological response at these sites that appears to be influenced by spill response efforts and geography. This apparent mitigation notably contrasts the fungal dominance at other locations; continued sampling will provide an alternate view of community shifts and long-term ecosystem recovery at less heavily impacted sites.

## Discussion

Little is known about seasonal community shifts in the Gulf of Mexico. Although lower overall meiofauna densities have been reported in autumn months [16], low relative abundance of metazoan taxa in post-spill sediments is quite surprising. In addition to the inherent toxicity of hydrocarbon compounds, the unprecedented spill response effort likely had significant implications for microbial eukaryote communities. For example, at the time of sample collection the (closed) beach at Grand Isle, Louisiana was undergoing persistent, heavy oiling and a large-scale mechanical cleanup response (Figure S1). Within this impacted environment, sediment eukaryote communities were almost exclusively comprised of fungal taxa, with the proportion of *Cladosporium*-like OTUs representing >92% of environmental sequence reads. Although we cannot rule out temporal effects between our spring/autumn collection dates, the patterns of observed community shifts (phylogenetic analyses and relative abundance data), taxonomic biases in nematode genera, and metabolic capabilities of dominant post-spill fungal taxa all point towards significant impacts resulting from the DH oil spill. Continued sampling will be critical for assessing long-term trends and determining biological responses to oil exposure versus natural cycles in eukaryote assemblages. Finally, the observed fungal response to the Gulf oil spill further highlights the potential utility of these taxa in biological and biochemical remediation, given the seemingly ubiquitous ability of environmental fungal isolates to utilize hydrocarbon compounds [14].

Although we observed a dramatic change in community structure subsequent to summertime oiling, whether these shifts will be maintained over the longer term is unclear. There was little visual evidence of oil at the time post-spill samples were collected (two months after the Macondo leak was capped). This important observation provides a compelling case for sustained temporal sampling to elucidate the longevity of eukaryote community shifts. For nematodes, the abundance of juvenile life stages in several post-spill samples could suggest either some degree of community recovery or developmental hindrances that prevented indviduals surviving to adulthood. Regardless, hydrocarbon-associated fungal communities notably dominated sediment eukaryote communities despite the cessation of surface oil slicks by the time of autumn sampling. Much evidence of taxonomic loss was also apparent in post-spill metazoan communities: prolonged community biases across microbial eukaryote communities (whether due to slow biological recovery or the persistent impacts of dispersed oil) could eventually translate into long-term effects for higher-level predators and food webs in Gulf ecosystems.

## Materials and Methods

Raw pyrosequencing data obtained from marine sediments have been deposited in Dryad (http://dx.doi.org/10.5061/dryad.4sd51d4b), MG-RAST (Submission ID: 4478931.3), and the NCBI SRA (Accession No. SRA050276.2).

### Sample Sites

Sediment samples were collected from locations at Dauphin Island and Mobile Bay, Alabama, prior to (May 2010) and following beach oiling (Sept 2010) as a result of the *Deepwater Horizon* oil spill; Grand Isle, LA was additionally sampled during ongoing heaving oiling in September 2010 (Figure S2 and Table S1). No specific permits were required for the described field studies, as sediment collections were overseen and managed through Auburn University. Collection locations were not privately owned or protected, and sampling did not involve endangered or protected species. Sediment was immediately frozen upon collection (molecular replicates) or preserved in DESS [17] (morphological replicates). The meiofauna fraction of all samples was subsequently extracted in Instant Ocean® (artificial seawater prepared as per manufacturer instructions) according to standard protocols [18] for decantation using a 45 µm sieve. Nematode specimens were subsequently removed from morphological replicates and identified down to genus.

### DNA Extraction, PCR Amplification and Sequencing

Per sample, environmental DNA was extracted from 200 µl of sediment via bead beating using a Disruptor Genie (Zymo Research, Orange, CA). Two diagnostic regions of the 18 S gene were amplified from environmental extracts using MID-tagged eukaryotic primers SSU_F04/SSU_R22 [19] and NF1/18 Sr2b [20]. Reactions (50 µl) were carried out using 2 µl or 4 µl of environmental genomic templates, 0.4 µM of each primer (Integrated DNA technologies, Coralville, IA, USA), 2.5 µl 10X DyNAzyme EXT Buffer containing MgCl_2_ (final reaction volume 1.5 mM MgCl_2_), 0.5 µl dNTP mix containing 10 µM each nucleotide, and 0.5 µl DyNAzyme EXT DNA polymerase (New England Biolabs) under the following reaction conditions: 95°C for 2 min followed by 30–35 cycles of denaturation at 95°C for 1 min, annealing at 50°C for 45 sec, extension at 72°C for 3 min, with a final extension of 72°C for 10 min. All PCR products were visualized on a 1.5% agarose gel containing Ethidium Bromide. Amplicons were purified using SPRI purification followed by QIAquick PCR purification (QIAGEN), and equimolar concentrations of all samples were submitted for sequencing. Sequencing was carried out on the GS FLX Titanium platform, with reads averaging 350–450 bps in length.

### Processing of Raw Pyrosequencing Reads

Raw sequence reads were processed and clustered within the QIIME pipeline [6], returning 939,806 de-multiplexed reads (>200 bp) after quality checks and trimming. Reads were subsequently separated according to gene region and analysed in parallel; both loci were independently seeded and clustered into Operational Taxonomic Units (OTUs) with UCLUST using pairwise sequence identity cutoff values of 95–99%. Alternative read filtering and clustering was carried out via the OCTUPUS pipeline [21] to compare and confirm ecological inferences from UCLUST data. In each independent workflow, Chimeric OTUs were separately identified and removed via the Blast Fragments approach and chimera.pl script in QIIME and OCTUPUS, respectively. In addition, a subset of reads from four sites (Shellfish Lab Post-spill, Belleair Blvd Post-spill, GrandIsle Post-spill, Belleair Blvd Pre-spill) were denoised with AmpliconNoise followed by chimera removal in Perseus [4] before clustering. Linux shell scripts were written to accommodate multiplexed sequence data and wrap the standard AmpliconNoise scripts into a single pipeline. Denoising our full 454 dataset was not computationally possible, since denoising millions of reads is extremely computationally intensive, and was unfeasible even on a high-memory server with 32 GB RAM). Instead, a subset of sites was used to overcome these significant computational limitations and confirm that “noisy” data was not skewing data analyses. Taxonomic assignments were generated for representative OTU sequences using MegaBLAST (megablast -d database path -D 2 -p 90 -a 2 -b 1 -v 1 -i infile -F F > outfile) to retrieve the top-scoring hit from GenBank’s non redundant nucleotide database. OTU sequences that did not return any significant hits (<90% sequence identity) were labelled as ’no match’.

### Diversity Analyses

Alpha and Beta diversity analyses (rarefaction, PCoA, Jackknife Cluster Analysis) were carried out in the QIIME pipeline [6]. Representative OTU sequences were aligned to release 104 of the SILVA [22] database (condensed and filtered at 97% identity) using PyNAST [23], followed by construction of minimum evolution phylogenies in FastTree [24]. Rarefaction analyses (Figure S3) were carried out using pseudoreplicate OTU datasets containing between 10 and 57967 sequences (in steps of 5795) with 10 repetitions performed per pseudoreplicate. PCoA and Jackknifed cluster analyses were both run using the Unifrac distance metric [5] on weighted datasets (normalized abundance values) subsampled to an even depth across sample sites. Phylogenetic examination of fungal OTUs was carried out manually in ARB, whereby representative OTU sequences were aligned via the SINA aligner and subsequently inserted into the SILVA eukaryotic guide tree (release 106) using Fast Parsimony Insertion (default parameters). Alignments were manually examined for quality and phylogenetic placement of OTUs was used to infer taxonomy. Morphological community analysis of nematode genera was performed using multivariate statistics in the software package PRIMER™ version 6 [25]. Bray-Curtis similarity was calculated on the abundance matrix after presence-absence transformation.

## Supporting Information

Figure S1 **Eukaryotic sediment community at Grand Isle, LA in September 2010.** Eukaryotic community assemblage dominated by fungal taxa during beach oiling in Autumn 2010; Taxonomic proportions inferred from non-chimeric, denoised 454 reads [4] clustered at 99% identity in UCLUST. Photos illustrate beach conditions at Grand Isle, LA at the time of sample collection.
(PDF)

Figure S2 **Map of sampling locations.** Locations of collection sites in Grand Isle, LA, Mobile Bay and Dauphin Island, AL. Approximate shoreline oiling is shown as cumulative observed data (grayscale, number of days oiled) and NESDIS radar anomaly analysis (green hashes) obtained from NOAA (http://www.geoplatform.gov/gulfresponse).
(PDF)

Figure S3 **Rarefaction analysis of community diversity.** Rarefaction plots generated in QIIME using Chao1 and Observed Species metrics, displayed for both pre-spill (solid lines) and post-spill (dashed lines) samples. Plots represent amplicons generated from the F04/R22 primer set; rarefaction analyses corresponded between both 18 S rRNA loci.
(PDF)

Table S1 **Sample metadata.** GPS coordinates, collection date, and number of high-quality sequence reads per primer (>200 bp) obtained from each sample.
(PDF)
