# Supplementary material for: Dramatic Shifts in Benthic Microbial Eukaryote Communities following the Deepwater Horizon Oil Spill
Source: PLoS One. 2012 Jun 6;7(6):e38550. doi: 10.1371/journal.pone.0038550 (PMC3368851; doi:10.1371/journal.pone.0038550)

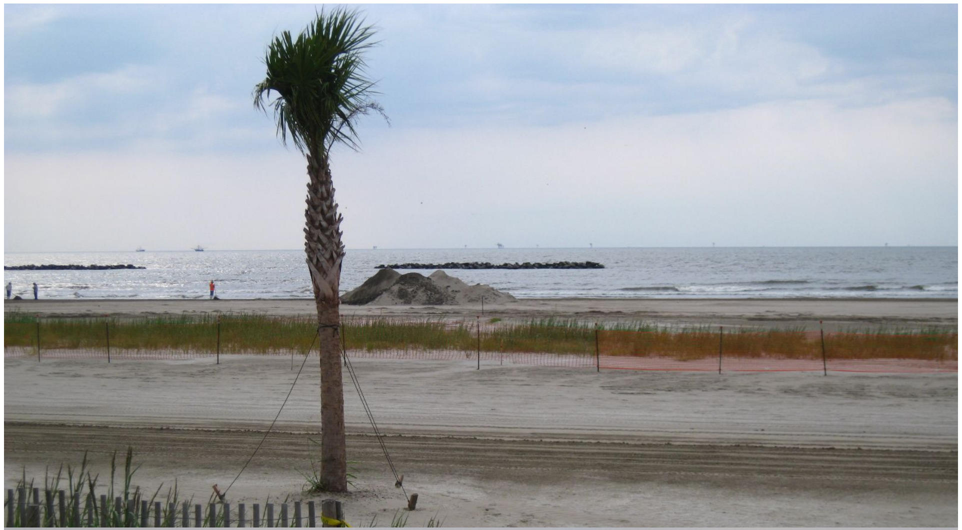

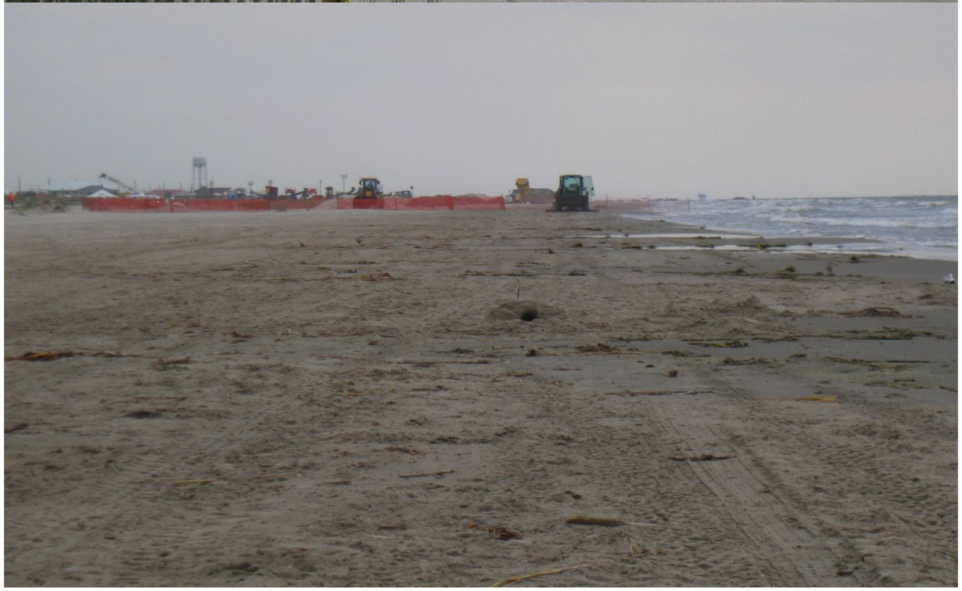

Post-Spill Sediment Community Grand Isle, LA

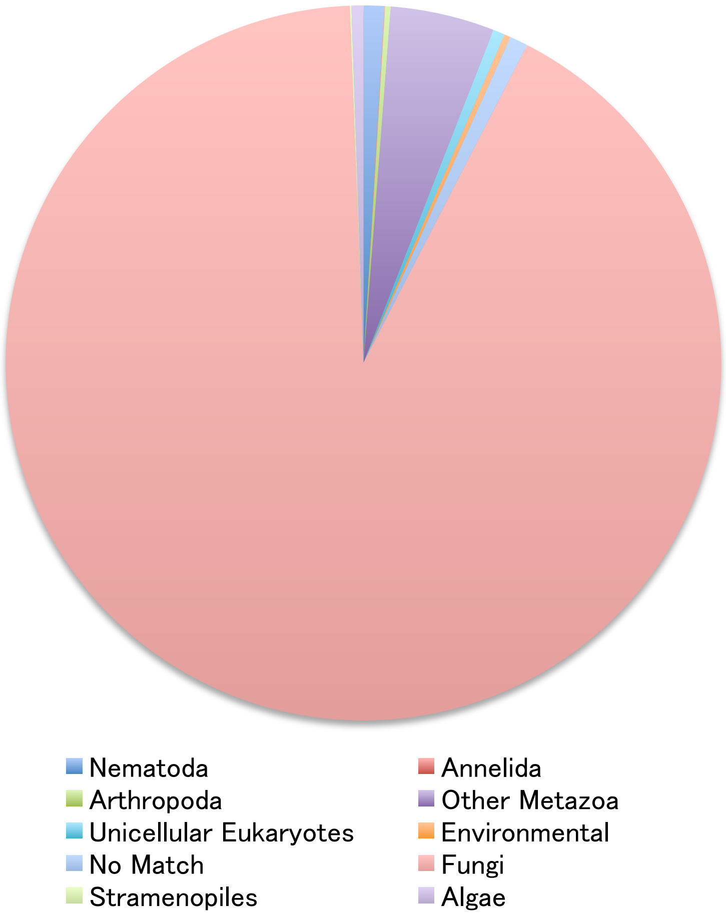

Supplement: Figure S1 — Eukaryotic sediment community at Grand Isle, LA in September 2010. Eukaryotic community assemblage dominated by fungal taxa during beach oiling in Autumn 2010; Taxonomic proportions inferred from non-chimeric, denoised 454 reads [4] clustered at 99% identity in UCLUST. Photos illustrate beach conditions at Grand Isle, LA at the time of sample collection. (PDF) [file pone.0038550.s001.pdf]
