# Supplementary figures and images for: Dramatic Shifts in Benthic Microbial Eukaryote Communities following the Deepwater Horizon Oil Spill
Source: PLoS One. 2012 Jun 6;7(6):e38550. doi: 10.1371/journal.pone.0038550 (PMC3368851; doi:10.1371/journal.pone.0038550)

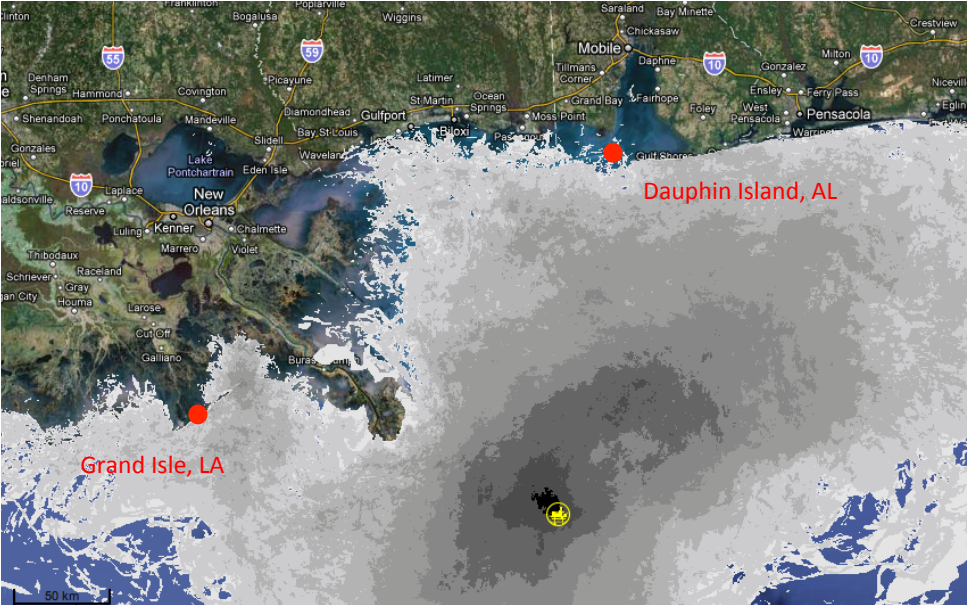

Cumulative oiling  
(days of oiling)

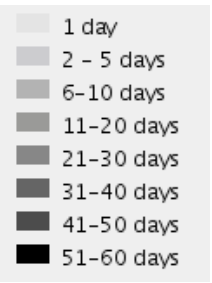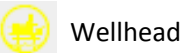

Wellhead

Cumulative NESDIS  
anomaly analysis  
(Apr-Aug 2010)

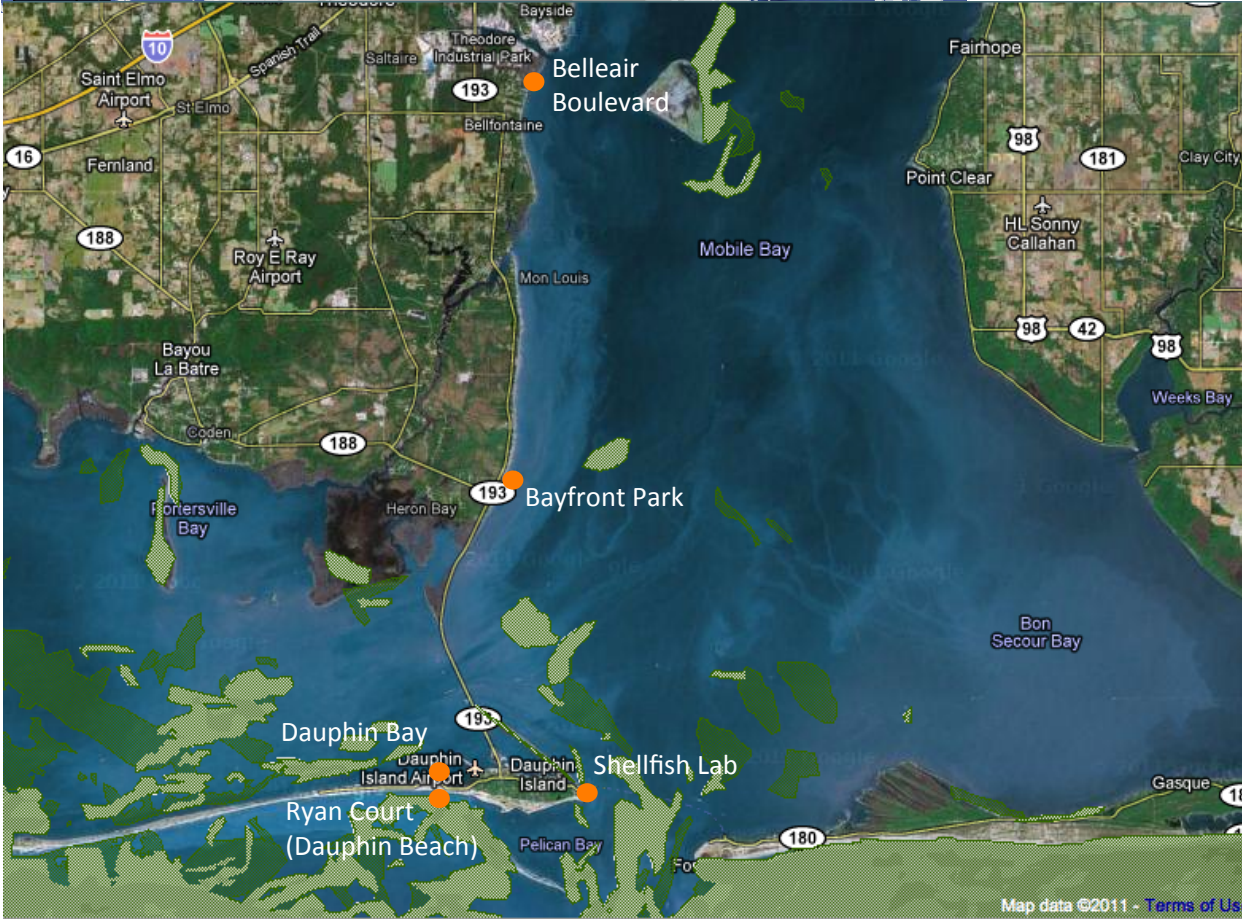

Supplement: Figure S2 — Map of sampling locations. Locations of collection sites in Grand Isle, LA, Mobile Bay and Dauphin Island, AL. Approximate shoreline oiling is shown as cumulative observed data (grayscale, number of days oiled) and NESDIS radar anomaly analysis (green hashes) obtained from NOAA (http://www.geoplatform.gov/gulfresponse). (PDF) [file pone.0038550.s002.pdf]

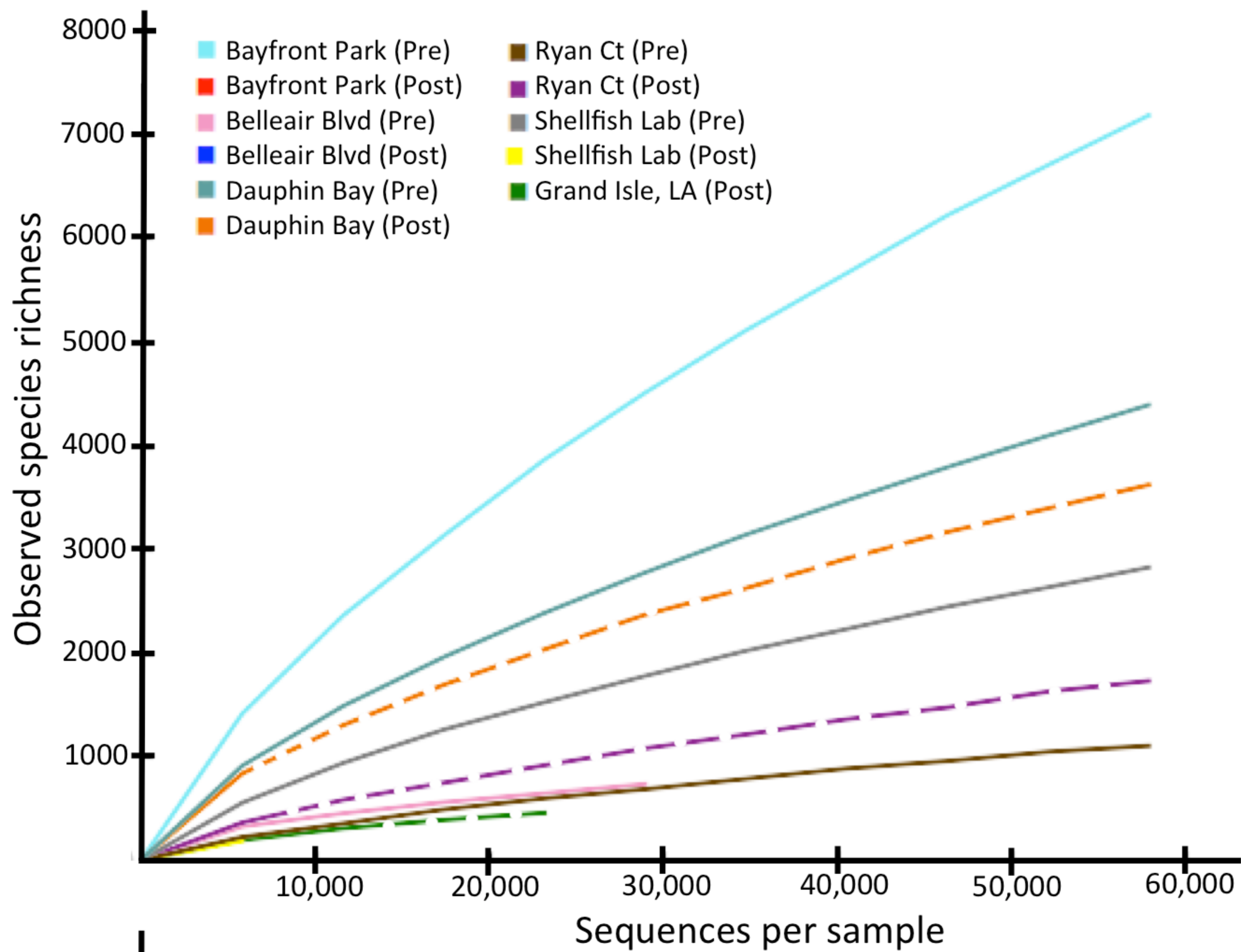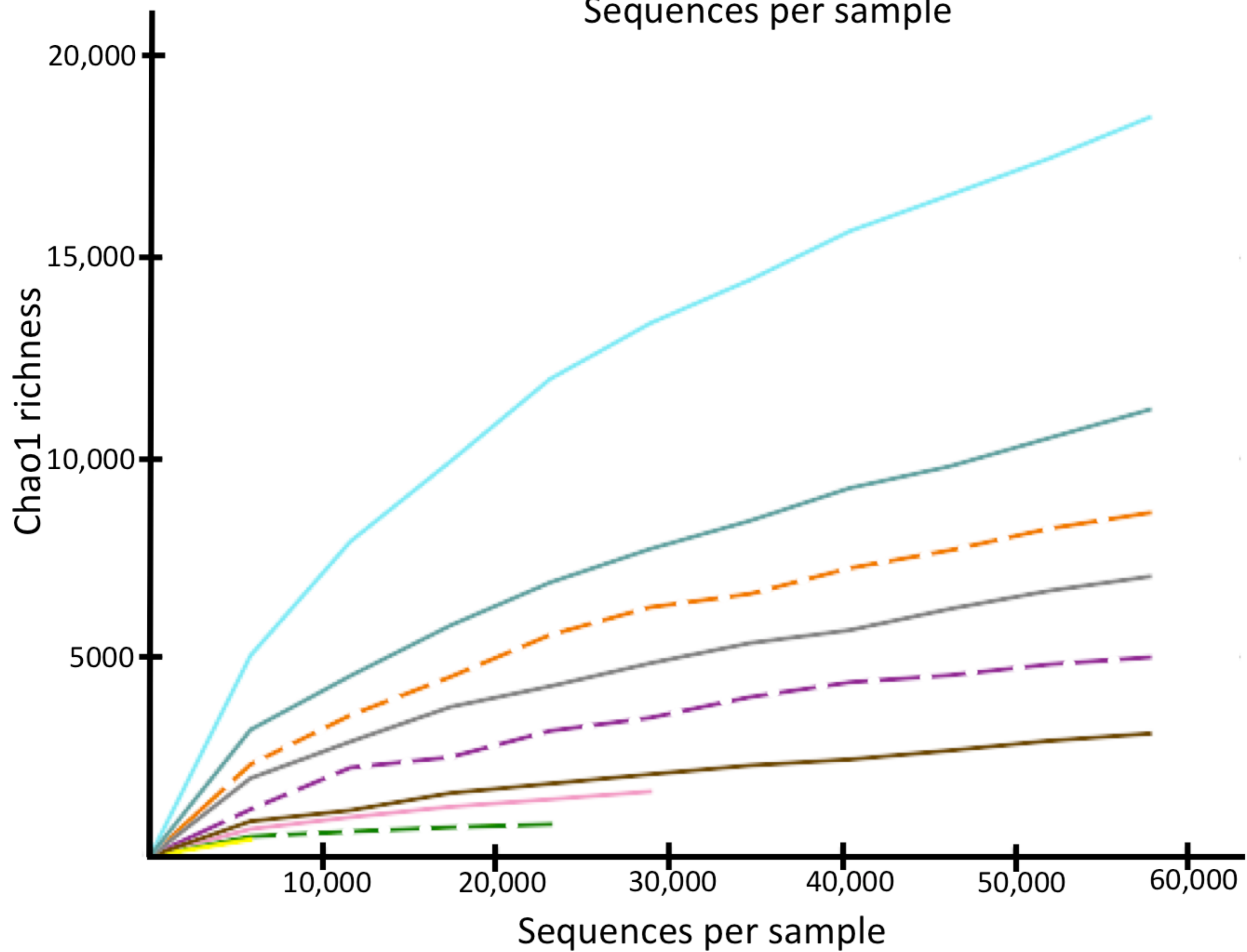

Supplement: Figure S3 — Rarefaction analysis of community diversity. Rarefaction plots generated in QIIME using Chao1 and Observed Species metrics, displayed for both pre-spill (solid lines) and post-spill (dashed lines) samples. Plots represent amplicons generated from the F04/R22 primer set; rarefaction analyses corresponded between both 18 S rRNA loci. (PDF) [file pone.0038550.s003.pdf]
