# Supplementary material for: Dramatic Shifts in Benthic Microbial Eukaryote Communities following the Deepwater Horizon Oil Spill
Source: PLoS One. 2012 Jun 6;7(6):e38550. doi: 10.1371/journal.pone.0038550 (PMC3368851; doi:10.1371/journal.pone.0038550)
Supplement: Table S1 — Sample metadata. GPS coordinates, collection date, and number of high-quality sequence reads per primer (>200 bp) obtained from each sample. (PDF) [file pone.0038550.s004.pdf]

Collection Date	Site	Latitude	Longitude	Condition	Reads per 18S primer F04	R22	NF1	18Sr2b
7-May-2010	Bayfront Park, AL	30° 21.235’ N	88° 7.071’ W	Pre-spill	37,007	35,875	35,996	32,446
7-May-2010	Ryan Ct (Dauphin Island Beach), AL	30°15.014’ N	88° 8.755 W	Pre-spill	77,149	71,588	25,879	22,990
7-May-2010	Dauphin Island Bay, AL	30° 15.203’ N	88° 8.813’ W	Pre-spill	15,084	15,716	29,532	25,934
8-May-2010	Belleair Boulevard, AL	30° 30.482’ N	88° 6.098’ W	Pre-spill	5,700	4,519	15,970	14,209
8-May-2010	Shellfish Lab, AL	30° 14.793’ N	88° 4.723’ W	Pre-spill	83,619	86,360	27,639	23,402
20-Sep-2010	Grand Isle, LA	29° 13' 30.1116" N	90° 0' 27.8418" W	Post-spill	2,529	1,795	13,528	11,041
21-Sep-2010	Belleair Boulevard, AL	30° 30' 28.9722" N	88° 6' 7.2576" W	Post-spill	897	796	674	636
21-Sep-2010	Bayfront Park, AL	30° 21' 15.61" N	88° 7' 3.80" W	Post-spill	385	256	696	513
21-Sep-2010	Shellfish Lab, AL	30° 14' 49.7508" N	88° 4' 31.1988" W	Post-spill	3,111	2,932	1,818	1,427
21-Sep-2010	Ryan Ct (Dauphin Island Beach), AL	30° 15' 1.7316" N	88° 8' 46.1436" W	Post-spill	31,507	31,232	27,975	26,306
21-Sep-2010	Dauphin Island Bay, AL	30° 15' 4.9824" N	88° 8' 6.6552" W	Post-spill	24,913	23,072	22,699	21,734
